# Predicting walking-to-work using street-level imagery and deep learning in seven Canadian cities

**DOI:** 10.1038/s41598-022-22630-1

**Published:** 2022-11-01

**Authors:** Dany Doiron, Eleanor M. Setton, Jeffrey R. Brook, Yan Kestens, Gavin R. McCormack, Meghan Winters, Mahdi Shooshtari, Sajjad Azami, Daniel Fuller

**Affiliations:** 1 Respiratory Epidemiology and Clinical Research Unit, Research Institute of the McGill University Health Centre, Montréal, QC Canada; 2 Geography Department, University of Victoria, Victoria, BC Canada; 3 Department of Chemical Engineering and Applied Chemistry, Dalla Lana School of Public Health, University of Toronto, Toronto, ON Canada; 4 Centre de Recherche en Santé Publique, École de santé publique de l’Université de Montréal, Montréal, QC Canada; 5 Department of Community Health Sciences, Cumming School of Medicine, University of Calgary, Calgary, AB Canada; 6 Faculty of Health Sciences, Simon Fraser University, Burnaby, BC Canada; 7 Department of Computer Science, University of Victoria, Victoria, BC Canada; 8 School of Human Kinetics and Recreation, Memorial University of Newfoundland, St. John’s, NL Canada; 9 Department of Community Health and Epidemiology, College of Medicine, University of Saskatchewan, Saskatoon, SK Canada

**Keywords:** Risk factors, Computer science, Scientific data

## Abstract

New ‘big data’ streams such as street-level imagery are offering unprecedented possibilities for developing health-relevant data on the urban environment. Urban environmental features derived from street-level imagery have been used to assess pedestrian-friendly neighbourhood design and to predict active commuting, but few such studies have been conducted in Canada. Using 1.15 million Google Street View (GSV) images in seven Canadian cities, we applied image segmentation and object detection computer vision methods to extract data on persons, bicycles, buildings, sidewalks, open sky (without trees or buildings), and vegetation at postal codes. The associations between urban features and walk-to-work rates obtained from the Canadian Census were assessed. We also assessed how GSV-derived urban features perform in predicting walk-to-work rates relative to more widely used walkability measures. Results showed that features derived from street-level images are better able to predict the percent of people walking to work as their primary mode of transportation compared to data derived from traditional walkability metrics. Given the increasing coverage of street-level imagery around the world, there is considerable potential for machine learning and computer vision to help researchers study patterns of active transportation and other health-related behaviours and exposures.

## Introduction

Street-level imagery is becoming ubiquitous, via proprietary sources such as Google Street View (GSV) and openly via crowd-sourcing efforts like Open Street Cam. These new ‘big data’ streams are offering unprecedented possibilities for developing health-relevant data on the urban environment^1,2^. With images extracted from GSV or other similar sources, deep learning algorithms can be trained to identify features within the urban environment such as vehicles, bicycles, buildings, vegetation, people, and sidewalks, which can themselves be turned into geospatial data. Such data can then be used to assess spatial variations in health-relevant urban characteristics such as the presence of sidewalks and parks, or neighbourhood greenness, or to predict other exposures such as air pollution, noise, and socio-economic status.

Applying deep learning algorithms to street-level imagery is an increasingly popular method to conduct neighbourhood environment audits or to derive exposure variables used in health research. An early focus of these emerging tools has been to make use of street-level imagery to characterize micro-scale urban environments conducive to active commuting and physical activity^3,4^. Street-level imagery has been shown to be an effective data source for auditing walkable streets^5^, predicting pedestrian volumes and neighbourhood walkability^4^, and identifying changes to the built environment such as implementation of traffic calming measures^6^. International studies have shown associations of urban features extracted from street-level imagery with walking and cycling patterns, as well as health indicators^7–10^. Few studies however have explored the relationship between features derived from street-level imagery and active commuting in Canada^11^, and no study to date has examined these relationship in multiple cities across a country with the diversity of Canada. Walking and cycling patterns in Canadian cities may be different compared to previously examined locations in the UK^9^, and the USA^10^, because of differing built environment characteristics, topography, and weather.

This manuscript has two objectives. First, to examine the associations between features of the urban environment derived from street-level images and walking-to-work rates from the Canadian Census. Second, to compare how GSV-derived features perform in predicting walk-to-work rates relative to more widely used walkability measures^12,13^. We hypothesize that neighbourhood features derived from street-level imagery using deep learning algorithms will be associated with spatial patterns of commuting by walking and that their predictive ability is comparable to area-level walkability metrics. Assessing these relationships can help inform how readily available imagery data can be used to derive geographically consistent metrics of the urban environment that are relevant to health.

## Materials and methods

### Study areas

Our study area included data from seven large cities from across Canada: Vancouver, Edmonton, Calgary, Winnipeg, Toronto, Montreal and Halifax. Study cities were selected to provide a good representation of Canadian urban environments in terms of population/size, geographic location, climate, city age, and urban form diversity. Municipal boundaries for the year 2015 were determined using the DMTI Spatial Inc. (Desktop Mapping Technologies Inc.) Municipal Amalgamation File (MAF)^14^.

### Urban features extracted from GSV images

The most recent Google Street View (GSV) images available between 2009 and 2017 were extracted by the Canadian Urban Environmental Health Research Consortium (CANUE)^15^ for each postal code within seven Canadian cities. Single-Link Indicators (SLI) from DMTI Spatial Inc. postal code locations circa 2015 were employed to identify the exact location at which to extract GSV images. For each postal code, the SLI is the geographic coordinate that best represents the location where the majority of the population lives within a postal code zone. In Canadian urban areas, postal codes (including residential and commercial addresses) typically correspond to one side of a city block or even a single building in densely populated areas. We extracted images at street locations closest to each postal code SLI x, y coordinate. For a given location, GSV captures 12 images. This includes six horizontal images that form a 360 degree view at that location and six images taken at a 60 degrees angle looking upwards, to create a 360 degree view of taller buildings. For this project, we only made use of the 6 horizontal images to avoid double counting of features since horizonal and 60 degree angle images overlap, and given that most of the features of primary importance are only visible at street level. The capture date of each image was also collected.

Two deep learning methods were used to extract urban features (e.g. persons, bicycles, buildings) from street-level images: (i) image segmentation (IS), which extracts the *percent pixel coverage* of features in an image, and (ii) object detection (OD), which extracts *counts* of features in an image. Both of these methods have been used in the past for research on neighbourhood design and built environment. Nguyen and colleagues made use of IS algorthims to quantify the level of street greenness and OD algorithms to identify the pressence of crosswalks and determine building types (single detached house vs other) to examine relationships with obesity and diabetes^7^ and a number of other health outcomes^8^ across the USA. Nagata et al. made use of IS in order to derive different urban form factors that can predict older adult’s leisure walking behaviour in Tokyo^16^. IS algorithms were also used by Yin and Wang to quantify the proportion of sky in GSV images, which was used to show inverse associations with pedestian counts in Buffalo, New York^16^. Li et al.^17^ and Cai et al.^18^ employed IS to estimated street-level greenness. Finally, OD methods have been shown to be a reliable approach to detect and count pedestrians with reasonable accuracy^4^.

**Figure 1:**
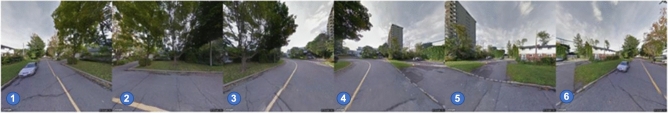
Example of six horizontal Google Street View images for a given postal code location*. *The image segmentation algorithm used all images (images 1 to 6) while the object detection algorithm used every second image (images 1, 3, and 5) to avoid counting the car twice. Images extracted from Google Street View: © 2002 Google.

We used freely available and powerful machine learning algorithms along with pre-trained datasets to derive urban features from GSV images. Pyramid scene parsing network (PSPNet)^19^ IS algorithms pre-trained using labelled images from the CityScapes dataset^20^ were employed to extract percent pixel coverage of features including persons, bicycles, buildings, sidewalks, open sky (without trees or buildings), and vegetation within GSV images corresponding to each postal code. You Only Look Once (YOLO) version 3^21^ OD algorithms pre-trained with the Common Object in Context (COCO) datasets^22^ were applied to extract counts of persons, bicycles and buildings for the same locations. Both PSPNet and YOLO models are well-documented and relatively easy to implement. In IS models, we made use of the six GSV images that look out horizontally for a given SLI x, y location (Fig. 1), while only three horizontal images (i.e. every second image) were used in OD models to reduce double-counting of objects. Images looking upwards at a 60 degree angle were not used in our analyses. We did not evaluate the accuracy of PSPnet or YOLO via comparison with our own ground truth images; however, PSPNet is reported to segment CityScape images with 80% accuracy, as indicated by the mean intersection over union (MIoU)^17^, and YOLO version 3 has a mean object precision (MaP) score of 58 when employed on the COCO image set, indicating that for detected objects (threshold set at 50% to confirm a positive object detection), the bounding box overlaps an average of 58% compared to ground truth bounding boxes^23^.

In total nine features were calculated for each postal code. With the IS method we calculated the number of pixels in each set of six images for: people [Person IS], bicycles [Bicycle IS], buildings [Building IS], sidewalks [Sidewalk IS], sky [Sky IS], and vegetation [Vegetation IS]. With the OD method, we used three images to calculate the count of: people [Person OD], bicycles [Bicycle OD], and buildings [Building OD]. In addition to data computed at the postal code SLI x, y coordinates, we calculated average percent pixel coverage and counts of each feature within all neighbouring postal codes in eight buffers of 250-m increments. Moving averages of each of the nine features listed above were therefore calculated in all postal codes x, y locations within buffer distances of 250, 500, 750, 1000, 1250, 1500, 1750 and 2000 m from the original observation. The distributions of Sky IS and Vegetation IS metrics were normally distributed. All other GSV image-derived metrics showed a right-skewed distribution. In order to assess correlations with walk commuting, we therefore log-transformed each remaining seven metric using log(x + 1) given the presence of zero values.

### Walkability data

The Canadian Active Living Environments (Can-ALE) dataset^12,24^ was used to compare the performance of features derived from street-level imagery in predicting walking commuting rates with that of pan-Canadian neighbourhood active living measures. The Can-ALE database, which is created using Geographic Information System (GIS)-based approaches, includes measures of intersection density, dwelling density, points of interest, and transit stops, which are features of the urban environment shown to be related to active transportation and physical activity patterns of Canadians. Further details on Can-ALE can be found elsewhere^25^. Each feature was calculated within one-kilometer circular buffers based on centroids of 2016 Canadian Census Dissemination Areas (DA) and calculated as z-scores. Dissemination Areas are geographic units composed of one or more adjacent city postal codes and the smallest geographic area for which all Canadian Census data are distributed. To allow analysis with features extracted from street-level images at the postal code, all single link postal code locations within a DA were assigned the same Can-ALE values.

### Commuting data

Walk-to-work rates were obtained from the 2016 Canadian Census. They correspond to the percentage of the working population using walking as their primary mode of transportation to reach their work destination. Statistics Canada reports census data at the DA level, which represents an average population of 400 to 700 individuals. As with Can-ALE data, we assigned walk commuting shares to all postal codes within each DA. There was a mean of 30 (SD = 47) postal codes per DA when considering data for all seven cities. Due to a strong right-skewed distribution, the walk-to-work rate variable was log-transformed for all postal codes in which some individuals reported walking to work (i.e. walk-to-work rate > 0%). DAs reporting zero percent of the population walking as the primary mode of transportation to work were not included in our analyses.

### Season

The image capture date was used to examine whether the season in which the image was taken modified the association between the percentage of people walking to work and the GSV features. We define seasons as winter (January, February, and March), spring (April, May, and June), summer (July, August, and September), and fall (October, November, and December).

### Statistical analysis

Statistical analyses proceeded in three main steps. First, for all seven cities combined, Pearson correlation coefficients were calculated for the log-transformed walk-to-work rates and all nine GSV features calculated (i) at postal code SLI x, y location and, (ii) for averages of GSV features within the eight predefined 250-m buffer distances from postal codes. Features with Pearson correlations of > 0.45 or < − 0.45 with walk-to-work data across all seven cities were subsequently used in regression models. For GSV features with strong correlations with log-transformed walk-to-work in analyses combining all cities, we also calculated Pearson correlations for each city separately in secondary analyses. Further given seasonal variations in the Canadian climate and its potential impact on the number ‘Person’ features we might see in GSV images throughout a year, calculations of Pearson correlations for log transformed ‘Person’ features with log walk-to-work rates were stratified by season.

Second, to assess the relative importance of GSV features and Can-ALE metrics in explained variance of log-transformed walk-to-work rates, we used linear regression and, given the structure of the data (mean of 30 postal codes per DA), Bayesian random intercept models implemented using R packages *lme4* and *brms*. The model for GSV features included a fixed effect for city along with ‘Person’ OD, ‘Building’ IS and ‘Sky’ IS, and their squared terms to account for potential non-linear associations^26^. The model for Can-ALE metrics included a city fixed effect variable, and street intersections, transit stops, dwellings and points of interests. In both models, variance explained of each predictor in log-transformed walk-to-work rates was estimated using R^2^ and 95% confidence intervals by sampling with replacement using 1000 bootstrap replicates. Individual and overall variance explained in log-transformed walk-to-work rates by the three GSV features and four Can-ALE metrics were calculated. Linear regressions with bootstrapping were conducted for all cities combined and for each city separately.

## Results

Table 1 presents descriptive statistics for the seven cities used in our analyses. The total number of postal codes in each city ranged from 11 410 (Halifax) to 85 536 (Toronto). Between 63.5% (Edmonton) and 73.7% (Halifax) of postal codes included some individuals reporting walking to work (e.g., non-zero walking to work mode share). For these postal codes, we downloaded a total of 1.15 million Google Street View images to be used in our analyses. City-specific counts of images ranged from 50 448 images in the city of Halifax to 341 772 images in the city of Toronto. Finally, the walk-to-work rate amongst postal codes with > 0% walking commuters ranged from 6.5% (Edmonton) to 13.5% (Halifax).

**Table 1:** Population, area, population density, number of census tracts, and number of street-level images.

	Vancouver	Edmonton	Calgary	Winnipeg	Toronto	Montreal	Halifax
Total population	2,463,431	1,321,426	1,392,609	778,489	5,928,040	4,098,927	403,390
Land area (km^2^)	2883	9439	5110	5307	5906	4604	5496
Population density (persons/km^2^)	854.6	140.0	272.5	146.7	1 003.8	890.2	73.4
Total number of dissemination areas	3430	1599	1721	1179	7368	6198	577
Total number of postal codes	59,800	31,684	29,238	17,868	85,536	46,214	11,410
Number of postal codes with > 0% walk-to-work rates (% of total)	41,580 (69.5)	20,126 (63.5)	20,338 (69.6)	12,542 (70.2)	56,962 (66.7)	31,625 (68.4)	8408 (73.7)
Number of street level images used	249,480	120,756	122,028	75,252	341,772	189,750	50,448
Walk-to-work (%)	8.43	6.51	8.07	8.34	7.53	8.06	13.5

**Figure 2:**
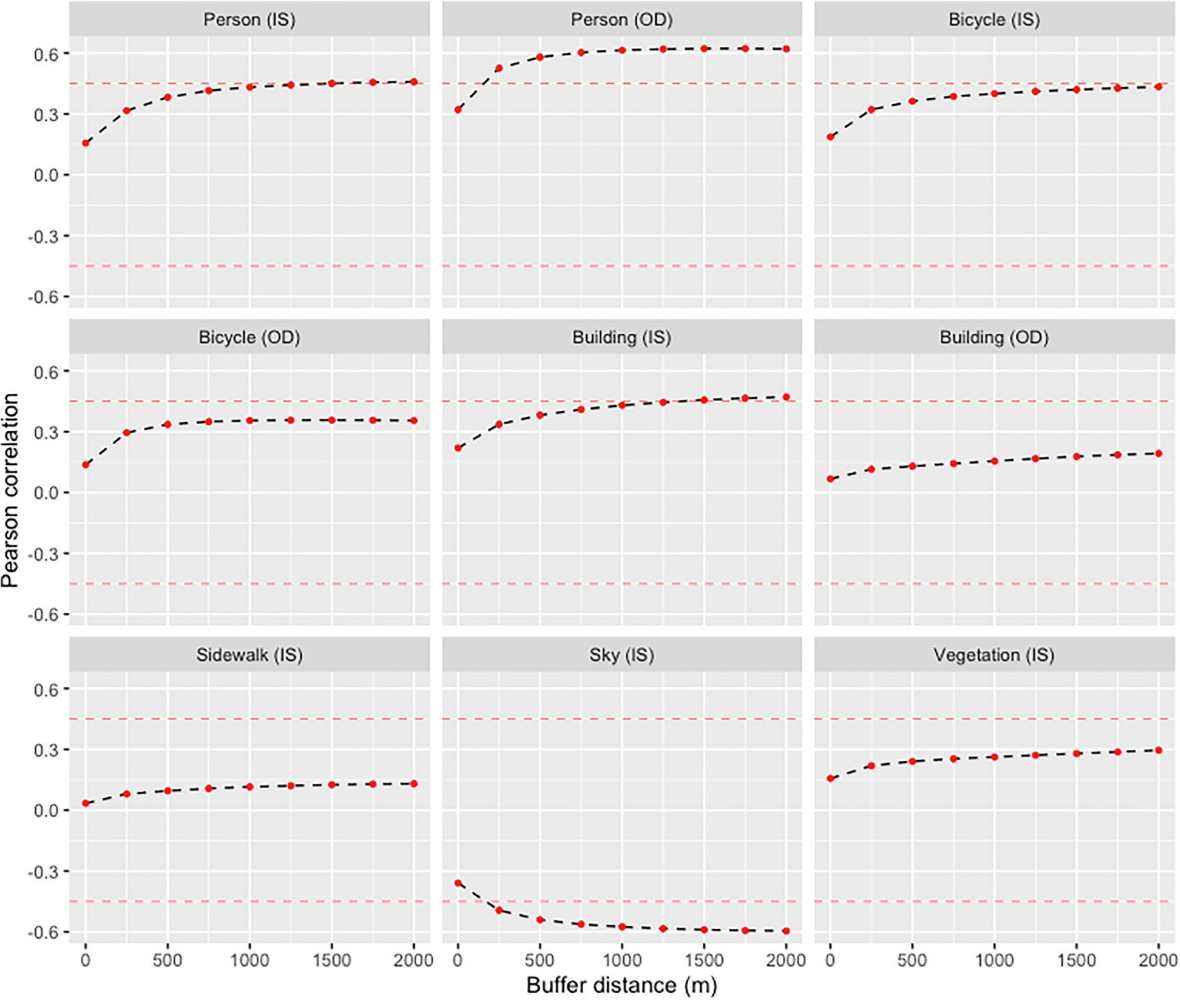
Pearson correlation coefficients for log-transformed walk-to-work rate and pixel coverage (IS) and counts (OD) of different features derived from GSV images within different buffer distances from postal codes*. *Walk-to-work rates are for postal codes with > 0% reported walk commuting. Horizontal dashed lines show > 0.45 and − 0.45 correlation thresholds used to identify variables for inclusion in subsequent regression analyses.

**Figure 3:**
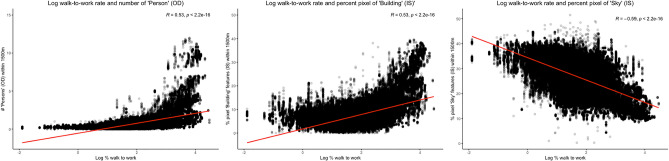
Linear relationships of GSV features within 1500 m from postal code with log-transformed walk-to-work rates.

Figure 2 shows Pearson correlation coefficients for the log-transformed walk-to-work rates and features derived from street-level images at postal code locations and averaged within 250-m buffer increments. For all features, the strength of correlations with log walk-to-work rates increased as values were averaged within increasing buffer sizes, up to 1500 m. Within 1500 m, Pearson correlation coefficients of > 0.45 or < − 0.45 were found for log ‘Person’ OD (R = 0.62) features, log ‘Building’ IS features (R = 0.46) and ‘Sky’ IS features (R = − 0.59) (Fig. 3). These three features were included in subsequent regression models. In city-specific correlation analyses for these pre-selected variables, considerable variation in correlation coefficients were found across the seven cities, with Pearson R ranging from 0.50 to 0.82 for log ‘Person’ OD (see Fig. S1 in Supplementary information file), 0.04 to 0.68 for log ‘Building’ IS (Fig. S2), and from -0.32 to -0.79 for Sky IS features (Fig. S3). When calculating correlations for log walk-to-work rates with log ‘Person’ features identified using object detection algorithms by season (Fig. S4), stronger correlations were seen for images taken in the winter (R = 0.83) compared to images taken in the other seasons (spring R = 0.65; summer R = 0.60; fall R = 0.63).

Table 2 shows the associations between individual features and log-transformed walking rates. The results of the linear models and Bayesian random intercept models are similar. All variables except ‘Building’ IS squared and ‘Sky’ IS squared were associated with log-transformed walking-to-work mode share. ‘Person’ OD had the strongest association with log-transformed walking rates in both linear and Bayesian random intercept models.

**Table 2:** Associations between individual GSV features and log-transformed walk-to-work rates, all cities combined.

*Predictors*	Linear model*	Bayesian random intercepts*
	*Estimates (95% CrI)*	*Estimates (95% CrI)*
Person 1500 OD	0.44 (0.43 to 0.45)	0.44 (0.44 to 0.45)
Person 1500 OD*2	− 0.03 (− 0.03 to − 0.03)	− 0.03 (− 0.03 to − 0.03)
Building 1500 IS	− 0.01 (− 0.01 to − 0.01)	− 0.01 (− 0.01 to − 0.01)
Building 1500 IS*2	0.00 (− 0.00 to − 0.00)	0.00 (− 0.00 to − 0.00)
Sky 1500 IS	0.02 (0.02 to 0.03)	0.02 (0.02 to 0.03)
Sky 1500 IS*2	0.00 (− 0.00 to − 0.00)	0.00 (− 0.00 to − 0.00)
**Random effects**		
σ^2^		0.07
τ_00_		0.00 _DA_uid_
ICC		0
N		14,330 _DA_uid_
Observations	191,581	191,581
R^2^/R^2^ adjusted	0.488/0.488	0.488/0.488

*Models included a fixed effect for each city. *OD *object detection, *IS *image segmentation, and *CrI *credible interval.

The variance explained in log-transformed walking rates by GSV features and Can-ALE metrics are presented in Table 3. ‘Person’ OD variables accounted for 14.4% of the variation in the proportion of individuals reporting walking to work, while ‘Building’ IS and ‘Sky’ IS accounted for an additional 11.9% and 19.7% of the variation in walk-to-work rates, respectively. In total, GSV-derived urban features with city-level adjustment accounted for 48.8% of the variation in walk-to-work rates for postal codes where at least some individuals walked to work (Table 3). In comparison, the combination of the city fixed effect variable, and Can-ALE metrics accounted for 39.8% of the variation in walk-to-work rates (Table 3). In city-specific analyses there was a considerable range in variance explained in log-transformed walking rates by both GSV features (Table S1a–g) and Can-ALE features (Table S2a–g). The cumulative adjusted R^2^ ranged from 41.3 to 76.6 for GSV features (Table S1a–g) and from 38.8 to 68.5 for Can-ALE metrics (Table S2a–g).

**Table 3:** Relative importance GSV features, Can-ALE metrics for log-transformed walk-to-work rates, all cities combined.

	Relative importance, adjusted R^2^ (95% CI)
**Percent of variation in walk-to-work rates explained by GSV features**	
City	2.8 (2.7, 3.0)
Person OD + Person OD^2^	14.4 (14.2, 14.6)
Building IS + Building IS^2^	11.9 (11.7, 12.1)
Sky IS + Sky IS^2^	19.7 (19.5, 19.9)
All factors combined	48.8 (48.1, 49.6)
**Percent of variation in walk-to-work rates explained by Can-ALE metrics**	
City	3.6 (3.4, 3.7)
Street intersections	6.3 (6.2, 6.4)
Transit stops	9.3 (9.1, 9.5)
Dwellings	9.5 (9.3, 9.6)
Points of interest	11.2 (11.0, 11.3)
All factors combined	39.8 (39.1, 40.4)

The R^2^ was calculated from linear regression models that included the variables indicated. The 95% confidence intervals of the R^2^ increments were estimated by sampling with replacement using 1000 bootstrap replicates. *OD *object detection, *IS* image segmentation, and *CI* confidence interval.

## Discussion

We examined the associations between features of the urban environment derived from street-level images using deep learning algorithms and walk-to-work rates obtained from the Canadian Census. We also compared the predictive ability of GSV features with that of a pan-Canadian dataset of metrics favoring active transportation. Our analyses showed that features derived from GSV images using deep learning algorithms can be used to explain variations in active transportation rates in areas of Canadian cities where commuting to work by walking is known to occur. When combined, ‘Person’, ‘Building’, and ‘Sky’ features derived using widely-available training datasets explained 48.8% of variations in walk-to-work rates in the seven Canadian cities included in our analyses, within postal codes in which at least some individuals reported commuting by walking. Together, dwelling density, transit stop density, street intersection density and points of interests, obtained from the Can-ALE dataset, explained 39.8% of walk-to-work variation. While the predictive power of GSV features relative to Can-ALE metrics was consistently stronger, there were considerable differences in variance explained of walk-to-work rates across the seven cities for each set of factors.

This research aligns with findings of previous studies conducted in other contexts. In a study conducted in the UK, Goel et al. (2018) found a moderate correlation between GSV images with pedestrians and walking for transportation in the past month (R = 0.46) and a weaker correlation for walking for any purpose in the past month (R = 0.3)^9^. The authors also reported a moderate correlation between the commute share of walking and GSV image observations of pedestrians (R = 0.43). In our study, we found correlations of R = 0.62 between the logged proportion of commuting done by walking and ‘Person’ features from GSV identified using object detection algorithms. Differences between the results could be partially explained by differences in the sampling of images and units of analysis. Goel et al. (2018) used 2000 images selected in 34 Primary Urban Areas in the UK, compared to 1.15 million images in 7 cities in our analyses. It is plausible that correlations would be weaker between a smaller sample of measures within a larger spatial unit of analysis. Broader physical differences between cities in Canada compared to those in the UK could also explain divergence in results. Associations between urban features and the proportion of people walking to work were consistent across cities in our study, although the strength of the associations varied between cities, in both correlation and linear regressions. Our results are consistent with previous research that has shown city specificity^7,8^, and suggests that transferability of models used to predict walking rates from one city to the other in Canada and internationally generalizability might be limited. Exploring the performance of other GSV-derived urban features in other contexts is warranted.

While a number of different features extracted from both image segmentation and object detection models have been used in the literature, it is still unclear which features are consistently associated with walking or physical activity. Using Google Street View images from the City of Buffalo, Yin et al. (2016) found positive associations between the proportion of sky extracted using machine learning and pedestrian counts^16^. Nguyen et al. (2021) used 31 M images from 2196 counties in the United States to show that the presence of non-single-family homes and single-lane roads was associated with greater physical inactivity^8^. Nguyen et al. (2018) used 430 000 images from Salt Lake City, Chicago and Charleston and showed that green streets, crosswalks and commercial buildings/apartments had relative obesity prevalence that were 25%-28% lower than individuals living in zip codes with the fewer of these urban features^7^. In Hong Kong, pixel coverage of street-level greenery extracted from Google Street View images was also associated with higher odds of walking and total walking time^27^. In our study, when combining data for seven cities strong correlations were seen for ‘People’, ‘Building’ and ‘sky’ features. However, the performance of each of these features in predicting walking varied considerably in city-specific analyses. Finally, for all cities combined, neither vegetation nor sidewalk pixel coverage were strongly correlated with walk commuting.

Our study has a number of strengths. By applying computer vision and deep learning techniques on a very large number of images obtained from GSV and multiple cities, we were able to assess predictors of walking to work rates across large geographic areas in an automated manner. As highlighted by previous work in this field, our results provide evidence of the potential for such technologies to assess active transportation rates in a rapid and cost-effective manner. Investigating these relationships across a diverse set of cities also enabled us to assess the generalizability of findings in different settings. The machine learning algorithms used in our study are freely available, have good accuracy, are well documented, and are relatively easy to implement. Training datasets employed for this project are also freely available and have been used elsewhere for similar purposes. The methods used in this study therefore facilitate replication of our work by other urban health researchers for other cities in Canada, and internationally.

There are a number of limitations to this work. First, because GSV images were obtained at the postal code level and walk-to-work rates and Can-ALE factors were obtained at the DA level, all postal codes within each DA were assigned the same walk-to-work and Can-ALE values. Second, spatial visualization and geostatistical analyses were not performed. We can therefore not link our results to geographic locations. Such analyses should be explored in future work ultimately to gain insight into patterns in walk-to-work that could inform future applications of imagery. Third, the outcome variable in our analysis is the percent of the population walking to work as their primary mode of transportation and was limited to areas where at least some people reported walking to work. Our study therefore did not allow us to infer relationships between GSV features and walking for reasons other than commuting to work, or for areas where no individuals walk to work. Getting a better sense of which urban features contribute to active living would require testing how features extracted from street-level images perform in predicting other purposes for walking, including errands or leisure. Unfortunately, Canada does not have a national travel survey, and thus such work could only be done where there are regional or municipal surveys with detailed information on active transportation behaviour broken down by age or other demographic variables, and ultimately, information that is relevant to physical activity levels associated with utilitarian and leisure-related transportation. Further, and as mentioned above, our methods for image detection can be used in other cities internationally, which would provide insights on the generalizability of associations in urban contexts beyond these seven cities. Finally, the use of street-level images to evaluate walking behaviour is limited by the types of features that can be captured. Many factors that might support walking behaviour (e.g., perceived safety, motor vehicle speeds) cannot be accurately detected by deep learning. Novel deep learning methods used to automatically detect and classify community amenities such retail stores from street-view imagery should also be explored to improve prediction of walking from GSV images^28^.

## Conclusion

This paper examined associations between walk commuting and features derived from image segmentation and object detection computer vision methods applied to Google street-level images in seven Canadian cities. Results showed that features derived from street-level images are better able to predict the percent of people walking to work as their primary mode of transportation compared to data derived from traditional walkability metrics such as Can-ALE. The results also showed city-level variations in associations between urban features and walk-to-work mode share. Given the increasing coverage of street-level imagery around the world, there is considerable potential for machine learning and computer vision to help researchers study patterns of active transportation and other health-related behaviours and exposures. Additional studies in other cities should be pursued to assess the feasibility of using GSV features derived from machine learning to estimate environmental exposures and health related behaviours such as active transportation in an automated and standardized manner. Successful implementation of such novel approaches to characterize the urban environment in different geographical contexts has considerable potential to lead to harmonized environmental metrics that can be used for health studies and for surveillance purposes such as tracking trends and comparing different geographic areas in terms of urban environment characteristics, and for epidemiological research. This information may ultimately direct planners and local governments towards modifiable features in the urban environment that favourably influence physical activity and other health-related behaviours and exposures in the population.

## Supplementary Information

Supplementary Information.

## Data Availability

The GSV images underlying the results presented in the study are available via Google’s Street View Static API (see: https://developers.google.com/maps/documentation/streetview). Canadian Active Living Environments (Can-ALE) data are available for download from the Canadian Urban Environmental Health Research Consortium (CANUE) data portal at https://canuedata.ca/.
