# Supplementary material for: Predicting walking-to-work using street-level imagery and deep learning in seven Canadian cities
Source: Sci Rep. 2022 Nov 1;12:18380. doi: 10.1038/s41598-022-22630-1 (PMC9626470; doi:10.1038/s41598-022-22630-1)
Supplement: Supplementary file 1 — Supplementary Information. [file 41598_2022_22630_MOESM1_ESM.pdf]

Supplementary information

Predicting walking-to-work using *Google Street View* imagery and deep learning: a study of seven Canadian cities

Dany Doiron, Eleanor M. Setton, Jeffrey R. Brook, Yan Kestens, Gavin R. McCormack, Meghan Winters, Mahdi Shooshtari, Sajjad Azami, and Daniel Fuller

Figure S1: Linear relationships of average 'Person' OD features within 1500 meters from postal code with log walk-to-work rates: City-specific results

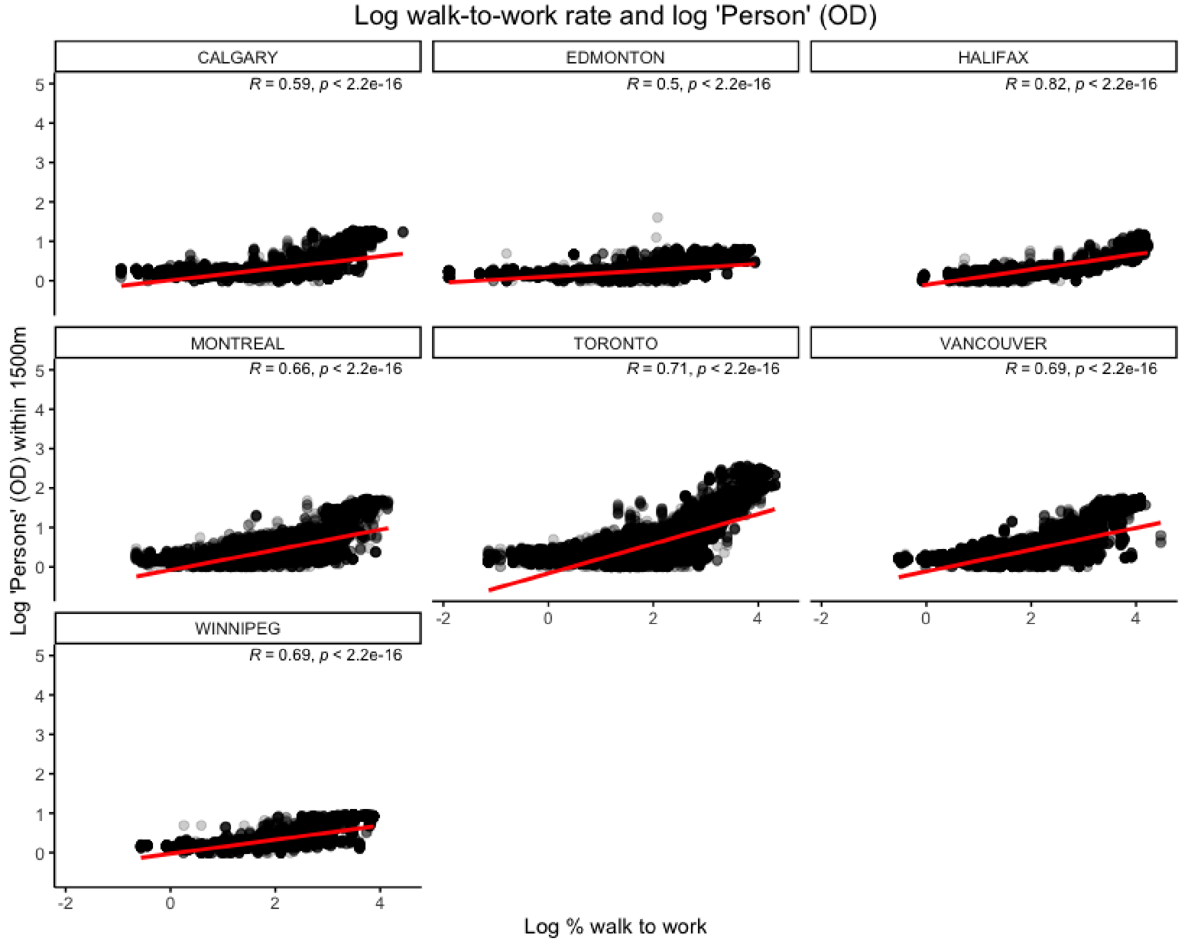

Figure S2: Linear relationships of average 'Building' IS features within 1500 meters from postal code with log walk-to-work rates: City-specific results

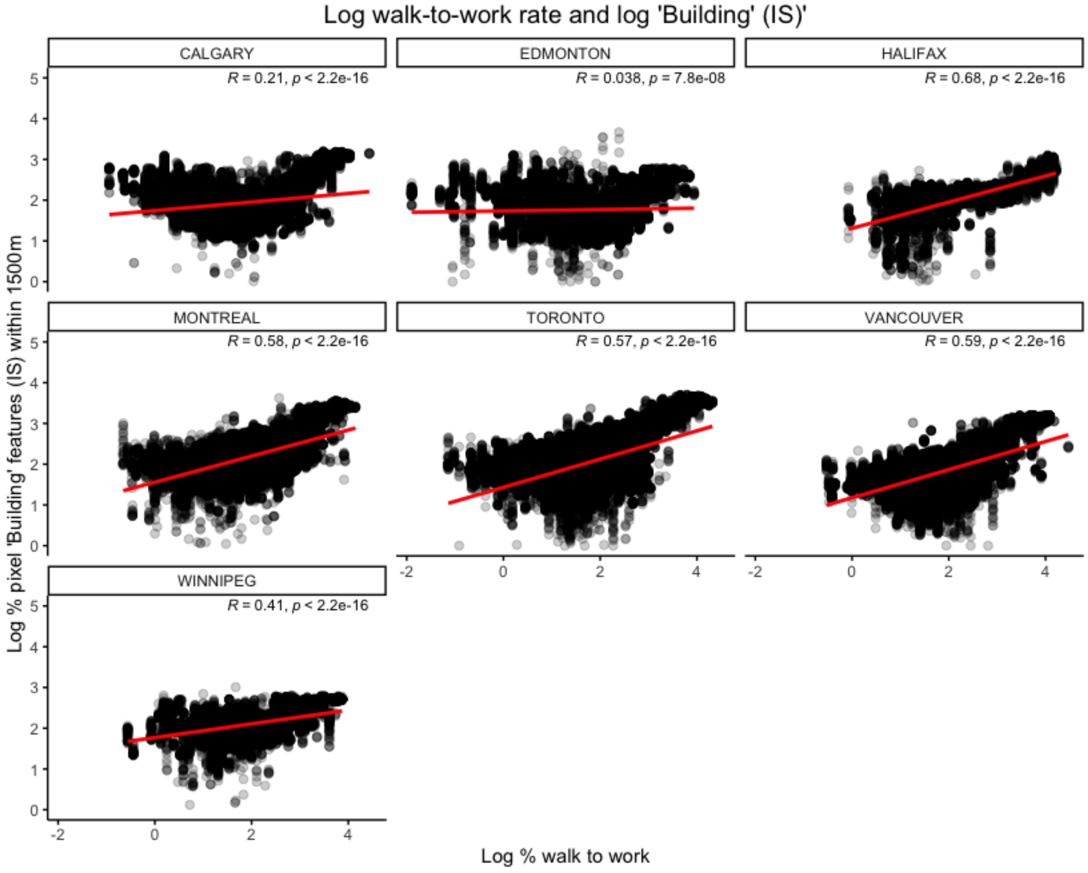

Figure S3: Linear relationships of average 'Sky' IS features within 1500 meters from postal code with log walk-to-work rates: City-specific results

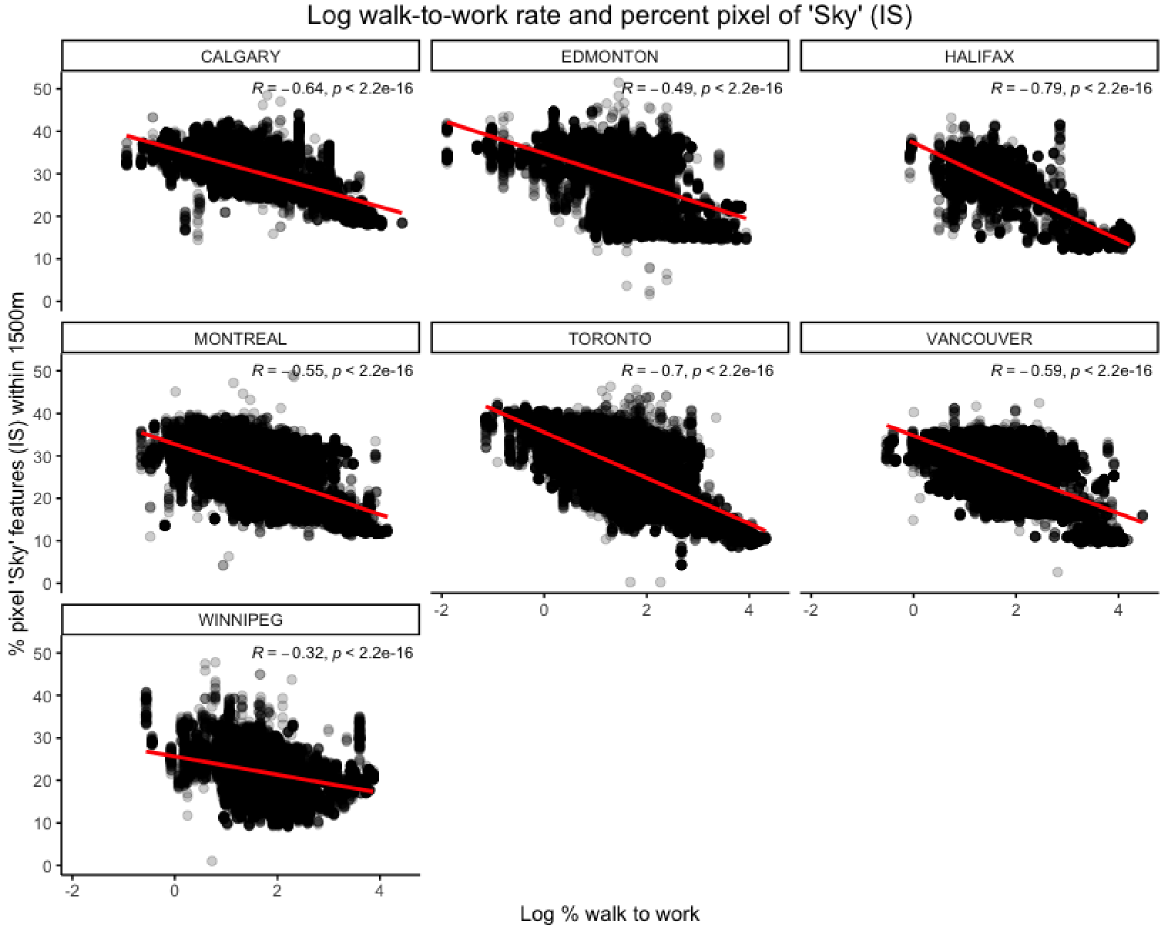

Figure S4: Linear relationships of average 'Person' OD within 1500 meters from postal code with log walk-to-work rates: results stratified by season

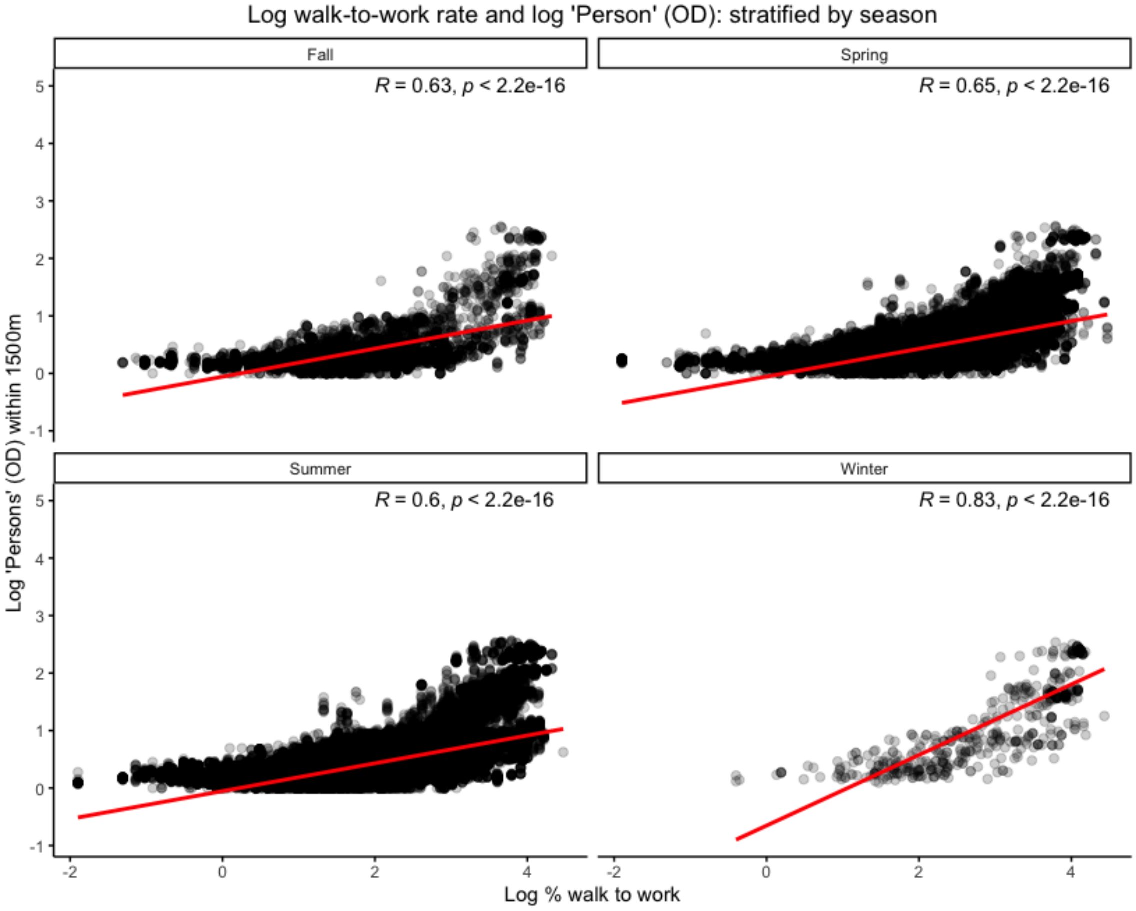

Table S1a: Variance explained in log-transformed walk-to-work rates by GSV features, city of Halifax

	**Relative importance, adjusted R^2 (95% CI)**
Percent of variation in walk-to-work rates explained, adjusted R^2	
Person OD + Person OD^2	23.9 (23.4, 24.5)
+ Building IS + Building IS^2	21.6 (21.0, 22.3)
+ Sky IS + Sky IS^2	31.1 (30.2, 31.9)
**All factors combined**	76.6 (74.6, 78.7)

The R^2 was calculated from linear regression models that included the variables indicated. The 95% confidence intervals of the R^2 increments were estimated by sampling with replacement using 1000 bootstrap replicates. Abbreviations: OD = object detection, IS = image segmentation, and CI = confidence interval. N = 8408

Table S1b: Variance explained in log-transformed walk-to-work rates by GSV features, city of Montreal

	Relative importance, adjusted R^2 (95% CI)
Percent of variation in walk-to-work rates explained, adjusted R^2	
Person OD + Person OD^2	17.0 (16.5, 17.4)
+ Building IS + Building IS^2	16.4 (15.8, 16.9)
+ Sky IS + Sky IS^2	12.8 (12.4, 13.4)
**All factors combined**	46.2 (44.7, 47.7)

The R^2 was calculated from linear regression models that included the variables indicated. The 95% confidence intervals of the R^2 increments were estimated by sampling with replacement using 1000 bootstrap replicates. Abbreviations: OD = object detection, IS = image segmentation, and CI = confidence interval. N = 31625

Table S1c: Variance explained in log-transformed walk-to-work rates by GSV features, city of Toronto

	Relative importance, adjusted R^2 (95% CI)
Percent of variation in walk-to-work rates explained, adjusted R^2	
Person OD + Person OD^2	17.4 (17.1, 17.7)
+ Building IS + Building IS^2	17.3 (17.1, 17.7)
+ Sky IS + Sky IS^2	24.2 (23.8, 24.7)
**All factors combined**	65.4 (64.3, 66.9)

The R^2 was calculated from linear regression models that included the variables indicated. The 95% confidence intervals of the R^2 increments were estimated by sampling with replacement using 1000 bootstrap replicates. Abbreviations: OD = object detection, IS = image segmentation, and CI = confidence interval. N = 56962

Table S1d: Variance explained in log-transformed walk-to-work rates by GSV features, city of Winnipeg

	Relative importance, adjusted R^2 (95% CI)
Percent of variation in walk-to-work rates explained, adjusted R^2	
Person OD + Person OD^2	31.4 (30.3, 32.5)
+ Building IS + Building IS^2	12.1 (11.3, 12.9)
+ Sky IS + Sky IS^2	5.8 (5.2, 6.5)
**All factors combined**	49.3 (46.8, 51.9)

The R^2 was calculated from linear regression models that included the variables indicated. The 95% confidence intervals of the R^2 increments were estimated by sampling with replacement using 1000 bootstrap replicates. Abbreviations: OD = object detection, IS = image segmentation, and CI = confidence interval. N = 12542

Table S1e: Variance explained in log-transformed walk-to-work rates by GSV features, city of Edmonton

	**Relative importance, adjusted R^2 (95% CI)**
Percent of variation in walk-to-work rates explained, adjusted R^2	
Person OD + Person OD^2	20.4 (16.7, 23.9)
+ Building IS + Building IS^2	3.9 (3.3, 4.5)
+ Sky IS + Sky IS^2	17.0 (16.1, 17.9)
**All factors combined**	41.3 (36.1, 46.3)

The R^2 was calculated from linear regression models that included the variables indicated. The 95% confidence intervals of the R^2 increments were estimated by sampling with replacement using 1000 bootstrap replicates. Abbreviations: OD = object detection, IS = image segmentation, and CI = confidence interval. N = 20126

Table S1f: Variance explained in log-transformed walk-to-work rates by GSV features, city of Calgary

	**Relative importance, adjusted R^2 (95% CI)**
Percent of variation in walk-to-work rates explained, adjusted R^2	
Person OD + Person OD^2	20.6 (20.0, 21.4)
+ Building IS + Building IS^2	10.3 (9.9, 10.6)
+ Sky IS + Sky IS^2	26.6 (25.7, 27.5)
**All factors combined**	57.5 (55.6, 59.5)

The R^2 was calculated from linear regression models that included the variables indicated. The 95% confidence intervals of the R^2 increments were estimated by sampling with replacement using 1000 bootstrap replicates. Abbreviations: OD = object detection, IS = image segmentation, and CI = confidence interval. N = 20338

Table S1g: Variance explained in log-transformed walk-to-work rates by GSV features, city of Vancouver

	**Relative importance, adjusted R^2 (95% CI)**
Percent of variation in walk-to-work rates explained, adjusted R^2	
Person OD + Person OD^2	18.7 (18.3, 19.1)
+ Building IS + Building IS^2	17.6 (17.3, 18.0)
+ Sky IS + Sky IS^2	13.6 (13.2, 14.0)
**All factors combined**	49.9 (48.8, 51.1)

The R^2 was calculated from linear regression models that included the variables indicated. The 95% confidence intervals of the R^2 increments were estimated by sampling with replacement using 1000 bootstrap replicates. Abbreviations: OD = object detection, IS = image segmentation, and CI = confidence interval. N = 41580

Table S2a: Variance explained in log-transformed walk-to-work rates by Can-ALE metric, city of Halifax

	**Relative importance, adjusted R^2 (95% CI)**
Percent of variation in walk-to-work rates explained, adjusted R^2	
+ Street intersections	16.8 (16.3, 17.3)
+ Transit stops	16.7 (16.2, 17.2)
+ Dwellings	16.6 (16.2, 17.1)
+ Points of interest	18.4 (17.8, 19.1)
**All factors combined**	68.5 (66.5, 70.7)

The R^2 was calculated from linear regression models that included the variables indicated. The 95% confidence intervals of the R^2 increments were estimated by sampling with replacement using 1000 bootstrap replicates. Abbreviations: OD = object detection, IS = image segmentation, and CI = confidence interval. N= 8408

Table S2b: Variance explained in log-transformed walk-to-work rates by Can-ALE metric, city of Montreal

	**Relative importance, adjusted R^2 (95% CI)**
Percent of variation in walk-to-work rates explained, adjusted R^2	
+ Street intersections	16.1 (15.7, 16.5)
+ Transit stops	6.5 (6.2, 6.8)
+ Dwellings	9.0 (8.8, 9.3)
+ Points of interest	14.4 (14.0, 14.8)
**All factors combined**	46.0 (44.7, 47.4)

The R^2 was calculated from linear regression models that included the variables indicated. The 95% confidence intervals of the R^2 increments were estimated by sampling with replacement using 1000 bootstrap replicates. Abbreviations: OD = object detection, IS = image segmentation, and CI = confidence interval. N= 31625

Table S2c: Variance explained in log-transformed walk-to-work rates by Can-ALE metric, city of Toronto

	**Relative importance, adjusted R^2 (95% CI)**
Percent of variation in walk-to-work rates explained, adjusted R^2	
+ Street intersections	12.1 (11.9, 12.4)
+ Transit stops	7.6 (7.4, 7.9)
+ Dwellings	13.4 (13.2, 13.7)
+ Points of interest	14.9 (14.7, 15.1)
**All factors combined**	48.0 (47.2, 49.1)

The R^2 was calculated from linear regression models that included the variables indicated. The 95% confidence intervals of the R^2 increments were estimated by sampling with replacement using 1000 bootstrap replicates. Abbreviations: OD = object detection, IS = image segmentation, and CI = confidence interval. N= 56962

Table S2d: Variance explained in log-transformed walk-to-work rates by Can-ALE metric, city of Winnipeg

	**Relative importance, adjusted R^2 (95% CI)**
Percent of variation in walk-to-work rates explained, adjusted R^2	
+ Street intersections	3.2 (2.9, 3.5)
+ Transit stops	14.6 (13.7, 15.5)
+ Dwellings	8.4 (7.9, 8.9)
+ Points of interest	20.1 (19.5, 20.7)
**All factors combined**	46.3 (44.0, 48.6)

The R^2 was calculated from linear regression models that included the variables indicated. The 95% confidence intervals of the R^2 increments were estimated by sampling with replacement using 1000 bootstrap replicates. Abbreviations: OD = object detection, IS = image segmentation, and CI = confidence interval. N= 12542

Table S2e: Variance explained in log-transformed walk-to-work rates by Can-ALE metric, city of Edmonton

	Relative importance, adjusted R^2 (95% CI)
Percent of variation in walk-to-work rates explained, adjusted R^2	
+ Street intersections	5.2 (4.8, 5.6)
+ Transit stops	4.8 (4.5, 5.1)
+ Dwellings	7.9 (7.5, 8.3)
+ Points of interest	22.4 (21.8, 22.9)
**All factors combined**	40.3 (38.6, 41.9)

The R^2 was calculated from linear regression models that included the variables indicated. The 95% confidence intervals of the R^2 increments were estimated by sampling with replacement using 1000 bootstrap replicates. Abbreviations: OD = object detection, IS = image segmentation, and CI = confidence interval. N= 20126

Table S2f: Variance explained in log-transformed walk-to-work rates by Can-ALE metric, city of Calgary

	Relative importance, adjusted R^2 (95% CI)
Percent of variation in walk-to-work rates explained, adjusted R^2	
+ Street intersections	10.2 (9.8, 10.7)
+ Transit stops	11.3 (10.9, 11.7)
+ Dwellings	7.2 (6.8, 7.5)
+ Points of interest	10.1 (9.7, 10.5)
**All factors combined**	38.8 (37.2, 40.4)

The R^2 was calculated from linear regression models that included the variables indicated. The 95% confidence intervals of the R^2 increments were estimated by sampling with replacement using 1000 bootstrap replicates. Abbreviations: OD = object detection, IS = image segmentation, and CI = confidence interval. N= 20338

Table S2g: Variance explained in log-transformed walk-to-work rates by Can-ALE metric, city of Vancouver

	**Relative importance, adjusted R^2 (95% CI)**
Percent of variation in walk-to-work rates explained, adjusted R^2	
+ Street intersections	5.4 (5.1, 5.6)
+ Transit stops	9.1 (8.7, 9.4)
+ Dwellings	12.6 (12.3, 12.9)
+ Points of interest	14.8 (14.4, 15.2)
**All factors combined**	41.9 (40.5, 43.1)

The R^2 was calculated from linear regression models that included the variables indicated. The 95% confidence intervals of the R^2 increments were estimated by sampling with replacement using 1000 bootstrap replicates. Abbreviations: OD = object detection, IS = image segmentation, and CI = confidence interval. N= 41580
